# The Factors Associated with Person-Centered Care Competence among Nursing Students

**DOI:** 10.3390/ijerph19052787

**Published:** 2022-02-27

**Authors:** Myoungsuk Kim

**Affiliations:** Department of Nursing, College of Nursing, Kangwon National University, Chuncheon-si 24341, Korea; cellylife@gmail.com; Tel.: +82-33-250-8877

**Keywords:** person-centered care, positive psychological capital, ego-resiliency, depression, nursing students

## Abstract

To improve person-centered care (PCC) competence among nursing students, various associated factors must be considered. This study aimed to identify the factors influencing PCC competence among South Korean nursing students, using a descriptive, cross-sectional design. Participants were recruited from three nursing colleges in South Korea using convenience sampling. Data were collected from 1 December 2020 to 31 January 2021, using structured self-report questionnaires. Demographic information, positive psychological capital, ego-resiliency, and depression of the participants were assessed. Descriptive statistics, independent *t*-tests, one-way analyses of variance, and hierarchical multiple regression analysis were used in statistical analysis. Participants with highly positive psychological capital (*r* = 0.509, *p* < 0.001) and high ego-resiliency (*r* = 0.480, *p* < 0.001) had very good PCC competence. The multiple regression analysis revealed that 30.1% of the variance in PCC competence is attributable to positive psychological capital and ego-resiliency (*F* = 34.59, *p* < 0.001, adjusted *R*^2^ = 0.301). These results highlighted the need for strategies to enhance psychological factors, such as positive psychological capital and ego-resiliency, that could boost PCC competence in nursing students.

## 1. Introduction

Person-centered care (PCC) is a concept developed from the person-centered theory of American psychologist Carl Rogers [1]. It is an approach that focuses on patients or clients, as opposed to their ailments, in response to evolving healthcare paradigms. PCC is a key element of the healthcare sector today [2]. It refers to a humanistic care approach that enables healthcare providers to provide holistic care to individual patients based on the essence of nursing, respecting patients’ rights, and allowing them to make decisions about their treatment [1,3]. The term PCC is often used interchangeably with patient-centered care. However, although both concepts comprise factors such as empathy, relationships, communication, respect, and decision-making, the goal of patient-centered care is to promote the recovery of functional life, whereas PCC aims to help the patient attain a meaningful life [4]. Today, the healthcare paradigm is shifting from a patient-centered approach to a person-centered approach, viewing patients as human beings with preferences and needs beyond their diseases [5]. Thus, PCC is a concept that expands upon patient-centered care by considering the overall lives of patients [4].

Previous studies have reported a significant relationship between PCC and its outcomes. First, patients receiving PCC showed improved health outcomes, such as less pain, improved physical functions, and enhanced activities of daily living, and were more satisfied with the care provided by their nurses [6]. Moreover, nurses and staff who provided PCC showed greater job satisfaction, lower burnout and stress, and enhanced quality of care [7,8,9]. As shown here, PCC is valuable and significant to both patients and care providers; therefore, it may be a key topic to address and advance in clinical and educational settings.

Several countries worldwide, including the United Kingdom, the United States, and Canada, actively conduct research on PCC and are continuously advancing its theory and practice [5]. In contrast, the healthcare paradigm in Korea is still predominantly disease-focused. It primarily focuses on resolving medical problems, leading to difficulties in implementing PCC.

Thus, it is important to implement systematic PCC education and training in the nursing college curriculum so that it is actively practiced by clinical nurses in the future [10]. In previous studies, empathic competence [11], professional nursing values [12], interpersonal competence [11], and critical thinking [13] have been identified as predictors of PCC in nursing students. To incorporate PCC in nursing curricula, various associated factors should first be investigated. Accordingly, this study examined the effects of positive psychological capital, ego-resiliency, and depression on PCC competence among nursing students.

Positive psychological capital refers to a positive psychological state in which an individual pursues personal growth. It is an overarching concept integrating four aspects of positive psychology, namely, self-efficacy, optimism, hope, and resilience [14]. Positive psychological capital has been reported to reduce stress, improve psychological wellbeing and physical health, and alleviate burnout in nurses [15,16]. Moreover, it has been identified as a significant predictor of satisfaction among nursing students and was also correlated with their professional nursing values [17]. Professional nursing values are a predictor of PCC competence in nursing students [12]; thus, positive psychological capital is predicted to affect PCC competence by reducing negative aspects and amplifying positive aspects.

Ego-resiliency is the ability to adapt successfully to an evolving or stressful environment through self-regulation and flexibility [18]. Ego-resiliency is used interchangeably with resilience. However, the two differ in that resilience is the dynamic process of achieving positive adaptation through overcoming severe adversity [19], whereas ego-resiliency is a personality trait of the individual [20]. A high level of ego-resiliency helps individuals to adjust their internal factors more positively and build strong interpersonal relationships [18,21]. Interpersonal competence is a key attribute required to practice PCC by fostering strong relationships with patients and other health professionals [22]. Furthermore, ego-resiliency enables individuals to confront stressful situations and find positive meanings from them, thereby helping them solve problems [21]. Thus, ego-resiliency is an important factor for nursing students to learn in nursing practicum—to deal with unexpected situations in a clinical setting. Moreover, ego-resiliency also influences professional nursing values [23], which is an influencing factor of PCC competence in nursing students [12]. Thus, ego-resiliency is predicted to affect PCC competence in nursing students.

A large meta-analysis showed a high prevalence of depression (34%) among nursing students [24]; another study reported that professional nursing training courses in the nursing department are more likely to provoke emotional stress and thus cause depression in nursing students compared with other departments’ students [25]. Nursing students’ stress or depression not only affect their personal wellbeing and academic performance, but also influence their communication with patients during clinical practice. Depression can aggravate interpersonal challenges [26]; because good interpersonal relationships are an essential component of PCC [4], depression could act as a hindrance to optimally performing PCC. Thus, it is necessary to identify whether depression does indeed affect PCC competence.

**Figure 1:**
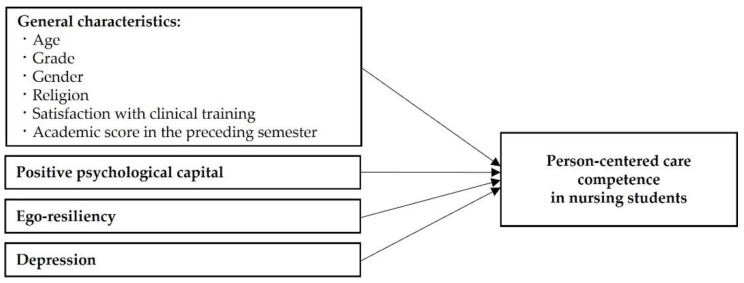
Conceptual framework for this study.

Positive psychological capital, ego-resiliency, and depression are believed to be important factors of PCC competence in nursing students; however, no previous study has investigated whether this is the case. This study aimed to confirm whether these variables are significant predictors of PCC competence in nursing students, ultimately enhancing PCC competence in them. We hypothesized that: (1) general characteristics would be an influential factor on PCC competence; (2) positive psychological capital would be an influential factor on PCC competence; (3) ego-resiliency would be an influential factor on PCC competence; and (4) depression would be an influential factor on PCC competence. The conceptual framework for this study is shown in Figure 1.

## 2. Methods

### 2.1. Aims

This study aimed to investigate the effects of general characteristics, positive psychological capital, ego-resiliency, and depression on PCC competence among South Korean nursing students.

### 2.2. Study Design

This study used a descriptive, cross-sectional design.

### 2.3. Participants

Participants were recruited from three nursing colleges in the Gangwon-do province of South Korea through convenience sampling, which was easily available to the researcher. The inclusion criteria were: (a) being a third- or fourth-year nursing student; and (b) having attended at least one semester of clinical training. The sample size was calculated using G*power 3.1.9 software (Heinrich-Heine-University, Düsseldorf, Germany) based on a significance level of 0.05, a power of 0.9, effect size of 0.15, and 9 associated variables. The minimum sample size was 152. The sample size was set to 170 considering a dropout rate of 12%. Out of 170, 169 questionnaires were collected; however, after excluding 12 questionnaires due to missing values, the final number of questionnaires was 157.

### 2.4. Data Collection

Participants were recruited using social network services and offline posters. Data were collected from 1 December 2020 to 31 January 2021, using structured questionnaires, by a trained research assistant. Data collection was carried out by research assistants unrelated to the research to minimize bias that could have occurred if the researcher conducted the questionnaire. In addition, in order to obtain accurate answers, participants were allowed to freely participate in the questionnaire at any time they wanted.

### 2.5. Measurements

#### 2.5.1. Demographic Characteristics

Age, grade, gender, religion, satisfaction with clinical training, and the preceding semester academic score of the participants were captured.

#### 2.5.2. PCC Competence

PCC competence was measured using the Individualized Care Scale-Nurse A version (ICS-A-Nurse) developed by Suhonen et al. [27]. The ICS-A-Nurse is for measuring individualized patient care from nurses’ perceptions, and includes needs, preferences, responsibility, and decision making [27], which are elements of person-centered care. This study used a version translated into Korean by Park [28]. The tool contains 17 items, each of which is scored on a five-point Likert scale, with higher scores indicating higher PCC. The reported content validity and item analysis in the study [27] were adequate. The internal consistency reliability in the original study was 0.88 [27]. In Park’s [28] study, the reliability using Cronbach’s alpha was 0.89; in this study, it was 0.87.

#### 2.5.3. Positive Psychological Capital

Positive psychological capital was measured using the Psychological Capital Questionnaire (PCQ) developed by Luthans et al. [29], revised for college students by Luthans et al. [30]. This study used a version translated into Korean by Kim [31]. The scale includes 24 items, with 6 items in each of the 4 sub-domains of self-efficacy, optimism, hope, and resilience. Each item is scored on 5-point Likert scale, with higher scores indicating a better positive psychological capital. The internal consistency reliability of this tool using Cronbach’s alpha was 0.91 in the study of Luthans, Youssef and Avolio [29], 0.90 in the study of Luthans, Luthans and Jensen [30], 0.93 in Kim’s study [31], and 0.89 in this study.

#### 2.5.4. Ego-Resiliency

Ego-resiliency was measured using the scale developed by Block and Kremen [18]. This study used a Korean version translated and revised by Yoo and Shim [32]. The tool includes 14 items, each of which consists of interpersonal relationships, curiosity, emotional control, vitality, and optimism. Each item is scored on 5-point Likert scale, with higher scores indicating better ego-resiliency. The internal consistency reliability by Cronbach’s alpha was 0.76 in the study of Block and Kremen [18], 0.67 in Yoo and Shim’s [32] study, and 0.75 in this study.

#### 2.5.5. Depression

Depression was measured using the integrated adaptation of CES-D (Center for Epidemiologic Studies Depression) Scale developed by Chon et al. [33] which enables the easy assessment of depressive symptoms in the general population. This 20-item tool uses a 4-point scale, ranging from 0 “rarely (≤1 day)” to 3 “most of the time (5–7 days).” The items concerning positive emotions are reverse-scored, which means that a higher score indicates more severe depression. The reported convergent validity and factor analysis in the study [33] are adequate. The internal consistency reliability of the scale using Cronbach’s alpha was 0.91 in Chon, Choi and Yang’s [33] study, and 0.81 in this study.

### 2.6. Ethical Considerations

This study was approved by the Institutional Review Board of the concerned University (IRB No. 2020-07-005-001) before the data collection. Prior to data collection, the participants were informed of the purpose and procedure of the study. They were assured of their anonymity and confidentiality, protection of personal information, data disposal, and freedom to withdraw from the study at any time. The participants voluntarily signed a written consent form and completed the questionnaires.

### 2.7. Statistical Analysis

Statistical analysis was conducted using IBM Statistical Package for the Social Sciences ver. 25.0 software (IBM Corp., Armonk, NY, USA). The participants’ general characteristics and main variables (PCC competence, positive psychological capital, ego-resiliency, and depression) were analyzed using descriptive statistics, including frequency with percentage, and the mean with standard deviation. The differences in PCC competence according to the general characteristics were analyzed using independent *t*-tests and one-way analyses of variance. The relationships among the main variables were identified by calculating Pearson’s correlation coefficients. Hierarchical multiple regression analysis was conducted to examine the factors influencing PCC competence. The level of significance was set to less than 0.05, and the reliability of the instruments was analyzed using Cronbach’s alpha.

## 3. Results

### 3.1. PCC Competence According to General Characteristics

The differences in PCC competence according to the participants’ general characteristics are described in Table 1. Most of the participants were under the age of 25 (83.4%), 60.5% were third-year students, and 86.6% were females. Most participants reported having no religion (58.6%). The majority (69.4%) reported being dissatisfied with clinical training, and most (64.3%) had an academic score of 3.0–3.9 (out of 4.5) in the preceding semester. In this study, there was no statistically significant difference in the mean PCC competence for age (*t =* −2.12, *p* = 0.097), grade (*t* = −0.86, *p* = 0.387), gender (*t* = 1.69, *p* = 0.092), religion (*t* = −0.11, *p* = 0.915), satisfaction of clinical practice (*t* = 1.18, *p* = 0.309), and academic score in the preceding semester (*t* = 0.69, *p* = 0.501).

### 3.2. Bivariate Relationships between PCC Competence and Associated Variables

Table 2 shows only the Pearson’s correlations between the main variables because there was no statistically significant difference in PCC competence according to the general characteristics. Participants with a highly positive psychological capital (*r* = 0.509, *p* < 0.001) and high ego-resiliency (*r* = 0.480, *p* < 0.001) demonstrated high PCC competence. Those with high ego-resiliency (*r* = 0.580, *p* < 0.001) and lower depression (*r* = −0.497, *p* < 0.001) had a high positive psychological capital. Participants with lower depression (*r* = −0.234, *p* = 0.003) had high ego-resiliency.

### 3.3. Factors Influencing PCC Competence

Table 3 summarizes the result of hierarchical multiple regression analysis for PCC competence. The homogeneity, multicollinearity of the residuals, and normal distribution were checked. The range of tolerance was 0.65–0.75, and that of the variation inflation factor (VIF) was 1.32–1.52, indicating no risk of multicollinearity between independent variables. Furthermore, the Durbin–Watson statistic for testing the independence of the residuals was 2.12, confirming no autocorrelation among the error terms.

The first model included positive psychological capital in the analysis. The positive psychological capital was a significant influencing factor of PCC competence (*F* = 54.29, *p* < 0.001). The first regression model explained 25.5% of the variance. Participants who had high positive psychological capital had higher PCC competence. The second model added ego-resiliency. Positive psychological capital and ego-resiliency were significant influencing factors of PCC competence (*F* = 34.59, *p* < 0.001). The second regression model explained 30.1% of the variance and had a large effect (effect size, f^2^ = 1.257). Participants who had high positive psychological capital and high ego-resiliency had higher PCC competence. Depression was added in the final model; it was not a significant influencing factor of PCC competence.

## 4. Discussion

This study investigated the effects of positive psychological capital, ego-resiliency, and depression on PCC competence among nursing students in South Korea. The findings of this study highlighted the need to consider positive psychological capital and ego-resiliency in interventions that aim to strengthen PCC competence in nursing students.

It was found that positive psychological capital is significantly positively correlated with PCC competence. Positive psychological capital has been reported to influence burnout among nurses [15], which can diminish their passion for their job. Additionally, positive psychological capital reduces burnout during clinical practice among nursing students. Moreover, positive psychological capital is one of the most important human capital subsets that can resolve people-related problems in an organization [14]. PCC requires continuous communication with the patient and active coordination with all healthcare and supportive service providers [34]. Therefore, positive psychological capital must be built and nurtured for nursing students who will become nurses in the future to improve their PCC competence. Furthermore, positive psychological capital was positively correlated with professional nursing values and satisfaction with their major among nursing students [17]. In particular, professional nursing values is the most influencing factor of PCC competence in nursing students [12], suggesting that positive psychological capital is a key factor in fostering PCC competence.

Ego-resiliency was significantly positively correlated with PCC competence in this study. Ego-resiliency has previously been reported as an influencing factor of professional nursing values in nursing students [23]. Professional nursing values are an influencing factor of PCC competence [12]; therefore, we can infer that ego-resiliency and PCC competence are significantly correlated. Specifically, ego-resiliency is one of the important factors of interpersonal competence among nursing students [35], and this interpersonal competence is an essential component of PCC competence [11]. Thus, boosting ego-resiliency is crucial for enabling nursing students to develop flexibility in adjusting to rapidly evolving or stressful situations in the hospital environment and strengthen their PCC competence.

In contrast, depression was not found to have a significant correlation with PCC competence. Previous studies found depression to be highly prevalent among nursing students [24], and those with depression have difficulties in maintaining interpersonal relationships [26], an important factor in PCC competence in nursing students [11]. However, nursing students may be subject to severe stress, and consequently develop depressive symptoms due to close patient contact and continuous emotional engagement. Thus, further studies are needed to develop insight into this topic.

Positive psychological capital and ego-resiliency were identified as influencing factors of PCC competence in nursing students in this study. This is an important finding, considering that no previous study has reported the effects of positive psychological capital and ego-resiliency on PCC competence among nursing students. These results show that these two factors must be considered when attempting to foster PCC competence among nursing students. Positive psychological capital was the most potent influencing factor of PCC competence. The four basic components of positive psychological capital—self-efficacy, hope, optimism, and resiliency—must be enhanced in order to build positive psychological capital in nursing students. Luthans and Youssef [36] proposed the following to increase positive psychological capital: first, self-efficacy can most effectively be boosted through mastery experiences, vicarious learning, social persuasion, and positive feedback; second, setting practical, specific, and measurable goals is important for individuals to acquire a sense of accomplishment to raise hope; third, optimism can be developed through an appreciation for present, practical, and flexible perspectives, and a lenient view of the past; finally, resiliency can be developed through readiness for crises to protect against all possible risks. However, specific education and training programs are required to help nursing students build positive psychological capital. Furthermore, it was found that positive psychological capital increased with improving ego-resiliency and decreasing depression in this study, and this should be considered when implementing new interventions to improve positive psychological capital.

Ego-resiliency was another influencing factor of PCC competence. Ego-resiliency facilitates interpersonal relationships [18,21], which are crucial to PCC competence [4]. Moreover, ego-resiliency influences professional nursing values, a critical influencing factor of PCC competence in nursing students [23], which may be the reason why ego-resiliency influences PCC competence. As shown here, ego-resiliency—the ability to flexibly cope with stress—should be improved to enhance PCC competence. Nursing students who chose a nursing major based on aptitude demonstrated the highest ego-resiliency compared with students who took up nursing simply as a job or through parental encouragement [23]. Thus, high schools should offer various activities to help students gain diverse experiences and check whether nursing is suitable for them. Furthermore, colleges should also offer various activities to allow students who have chosen nursing without knowing their aptitude to find specialties in nursing which are appropriate for them. Nursing students’ ego-resiliency was influenced by an internal health locus of control and self-esteem; therefore, instructors should provide positive encouragement and feedback to improve the student’s internal locus of control, and students, in turn, should increase their positive emotional experiences and self-confidence to enhance their self-esteem [37]. Moreover, based on this study’s results that ego-resiliency increased with decreasing depression among nursing students, interventions should aim to reduce depression in nursing students to boost their ego-resiliency.

These results indicate the need for strategies that strengthen positive psychological capital and ego-resiliency to improve PCC competence in nursing students. The significance of this study lies in the fact that it is the first to shed light on the importance of positive psychological capital and ego-resiliency on PCC competence among nursing students. Furthermore, these findings can be used to develop intervention programs that foster PCC competence in nursing students.

However, this study has several limitations. The participants were chosen through convenience sampling among nursing students from three universities in South Korea; therefore, the findings of this study have limited generalizability. Additionally, this study used a cross-sectional design, so causality cannot be established. Thus, relevant to the current study, to make the results more robust, future studies are needed to be replicated in samples from nursing students at diverse nursing schools. Furthermore, it is necessary to perform longitudinal studies to establish causality between variables in future studies. In addition, we recommend that future studies develop intervention programs aimed at improving PCC competence in nursing students with consideration to positive psychological capital and ego-resiliency and an assessment of their efficacy.

## 5. Conclusions

This study identified associated factors of PCC competence among nursing students. The factors influencing PCC competence in nursing students were identified as positive psychological capital and ego-resiliency.

Although this study has limitations due to its cross-sectional design, the results suggest that interventions to improve PCC competence among nursing students should include strategies to enhance positive psychological capital and ego-resiliency. However, the findings of this study should be applied with caution, because PCC competence in nursing students is affected by various variables beyond the noted psychological aspects (positive psychological capital and ego-resiliency). In addition, future studies should investigate additional factors affecting PCC competence in order to improve PCC competence in nursing students.

## Figures and Tables

**Table 1:** Difference in the PCC competence according to general characteristics (*n* = 157).

Characteristics	Categories	*n* (%)	Mean ± Standard Deviation	*t* or *F*	*p*-Value
Age (years)	<25	131 (83.4)	3.67 ± 0.43	−2.12	0.097
	≥25	26 (16.6)	3.88 ± 0.60		
Grade	Third year	95 (60.5)	3.68 ± 0.48	−0.86	0.387
	Fourth year	62 (39.5)	3.74 ± 0.47		
Gender	Female	136 (86.6)	3.68 ± 0.45	1.69	0.092
	Male	21 (13.4)	3.87 ± 0.56		
Religion	Yes	65 (41.1)	3.70 ± 0.49	−0.11	0.915
	No	92 (58.6)	3.71 ± 0.46		
Satisfaction with clinical practice	Satisfied	8 (5.1)	3.66 ± 0.35	1.18	0.309
	Neutral	40 (25.5)	3.61 ± 0.55		
	Dissatisfied	109 (69.4)	3.74 ± 0.44		
Academic score in the preceding semester	<3.0	12 (7.6)	3.76 ± 0.34	0.69	0.501
	3.0~3.9	101 (64.3)	3.73 ± 0.48		
	≥4.0	44 (28.0)	3.63 ± 4.87		

**Table 2:** Correlation between the main variables (*n* = 157).

Variables	PCC Competence	Positive Psychological Capital	Ego-Resiliency	Depression
	*r* (*p*-Value)	*r* (*p*-Value)	*r* (*p*-Value)	*r* (*p*-Value)
Positive psychological capital	0.509 (<0.001)	1		
Ego-resiliency	0.480 (<0.001)	0.585 (<0.001)	1	
Depression	−0.139 (0.083)	−0.497 (<0.001)	−0.234 (0.003)	1

PCC: person-centered care, tested using Pearson’s correlation coefficients.

**Table 3:** Hierarchical multiple regression of person-centered care competence (*n* = 157).

Variables	Model 1				Model 2				Model 3			
	*β*	*t*	*p*	95% CI	*β*	*t*	*p*	95% CI	*β*	*t*	*p*	95% CI
Positive psychological capital	0.509	7.368	<0.001	0.383~ 0.663	0.347	4.202	<0.001	0.189~ 0.524	0.419	4.543	<0.001	0.243~ 0.617
Ego-resiliency					0.277	3.360	0.001	0.099~ 0.381	0.266	3.231	0.002	0.089~ 0.371
Depression									0.132	1.711	0.089	−0.019~ 0.262
*F* (*p*)	54.29 (<0.001)				34.59 (<0.001)				24.32 (<0.001)			
*R* ^2^	0.259				0.557				0.568			
Adjusted *R*^2^	0.255				0.301				0.310			
Effect size (f^2^)	0.349				1.257				1.314			

CI: Confidence interval. As a measure of effect size, (f^2^) = 0.02, 0.15, and 0.35 represent small, medium, and large effects, respectively.

## Data Availability

The study data are available upon reasonable request from the corresponding author.
